# Antibody responses to the merozoite surface protein-1 complex in cerebral malaria patients in India

**DOI:** 10.1186/1475-2875-7-121

**Published:** 2008-07-04

**Authors:** Naomi W Lucchi, Jon Eric Tongren, Vidhan Jain, Avinash C Nagpal, Christian W Kauth, Ute Woehlbier, Hermann Bujard, Aditya P Dash, Neeru Singh, Jonathan K Stiles, Venkatachalam Udhayakumar

**Affiliations:** 1 Malaria Branch, Division of Parasitic Diseases, National Center for Zoonotic, Vector-Borne and Enteric Diseases, Coordinating Center for Infectious Diseases, Centers for Disease Control and Prevention, Atlanta, GA, USA; 2 National Institute of Malaria Research, Regional Medical Research Center for Tribals, Indian Council of Medical Research (ICMR), Jabalpur, India; 3 Nethaji Subash Chandra Bose (NSCB) Medical College, Jabalpur, India; 4 Zentrum fuer Molekulare Biologie Heidelberg (ZMBH), Universitaet Heidelberg, Im Neuenheimer Feld 282, D-69120 Heidelberg, Germany; 5 National Institute of Malaria Research, ICMR, New Delhi, India; 6 Morehouse School of Medicine, Atlanta, GA, USA; 7 Atlanta Research and Education Foundation, Decatur, GA, USA

## Abstract

**Background:**

*Plasmodium falciparum *infection causes cerebral malaria (CM) in a subset of patients with anti-malarial treatment protecting only about 70% to 80% of patients. Why a subset of malaria patients develops CM complications, including neurological sequelae or death, is still not well understood. It is believed that host immune factors may modulate CM outcomes and there is substantial evidence that cellular immune factors, such as cytokines, play an important role in this process. In this study, the potential relationship between the antibody responses to the merozoite surface protein (MSP)-1 complex (which consists of four fragments namely: MSP-1_83_, MSP-1_30_, MSP-1_38 _and MSP-1_42_), MSP-6_36 _and MSP-7_22 _and CM was investigated.

**Methods:**

Peripheral blood antibody responses to recombinant antigens of the two major allelic forms of MSP-1 complex, MSP-6_36 _and MSP-7_22 _were compared between healthy subjects, mild malaria patients (MM) and CM patients residing in a malaria endemic region of central India. Total IgG and IgG subclass antibody responses were determined using ELISA method.

**Results:**

The prevalence and levels of IgG and its subclasses in the plasma varied for each antigen. In general, the prevalence of total IgG, IgG1 and IgG3 was higher in the MM patients and lower in CM patients compared to healthy controls. Significantly lower levels of total IgG antibodies to the MSP-1_f38_, IgG1 levels to MSP-1_d83_, MSP-1_19 _and MSP-6_36 _and IgG3 levels to MSP-1_f42 _and MSP-7_22 _were observed in CM patients as compared to MM patients.

**Conclusion:**

These results suggest that there may be some dysregulation in the generation of antibody responses to some MSP antigens in CM patients and it is worth investigating further whether perturbations of antibody responses in CM patients contribute to pathogenesis.

## Background

One life-threatening complication of *Plasmodium falciparum *infection is cerebral malaria (CM). This complex syndrome affects mainly young children (two to six years old) in sub-Saharan Africa with an estimated incidence of 1.12 cases per 1,000 children per year and an estimated mortality of 18.6% [1]. In addition, a subset of CM survivors have an increased risk of developing persistent neurocognitive sequelae post-recovery [2-4] and reviewed in [5]. In Asia and South America, where the intensity of *P. falciparum *is much lower than in Africa, all age groups are at risk for CM [1,6-9]. The pathogenesis of CM is complex and it is still poorly understood as to why only a subset of patients develop CM. Various factors, such as sequestration of infected erythrocytes, and inflammatory cytokines and chemokines, have been postulated to play major roles in CM pathogenesis [10-17]. The role of antibodies in CM pathogenesis or protection is not well understood.

**Figure 1:**
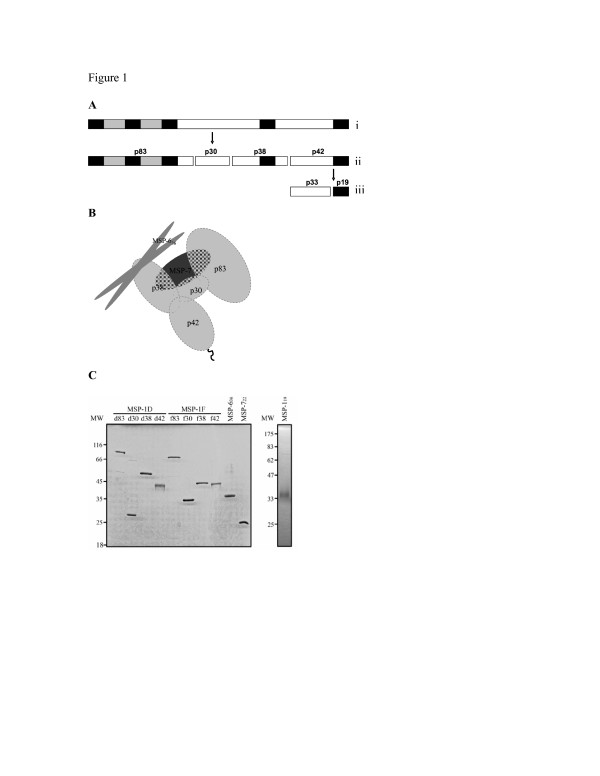
**Schematic representation of the MSP-1 and MSP-1/MSP-6/MSP-7 complex antigens used in the study**. The schematic representation of the MSP-1 protein is shown (A). The MSP-1 precursor protein (i) undergoes proteolytic cleavage into four subunits as shown (ii). The MSP-1_42 _molecule is further cleaved to MSP-1_33 _and MSP-1_19 _(iii). A proposed model (adapted from [23]) demonstrating the interactions of MSP-1 protein with the MSP-6_36 _and MSP-7_22 _molecules (B). The two allelic forms of MSP-1_83_, MSP-1_30_, MSP-1_38 _and MSP-1_42 _(D and F), in addition to MSP-6_36_, MSP-7_22 _and MSP-1_19_, were expressed in *E. coli *and purified [49,50]. The purity of these recombinant proteins was examined by using 12% SDS-PAGE followed by Coomassie staining (C). Molecular weight (MW) is shown in kDa.

The merozoite surface protein (MSP)-1, a large multiprotein complex exposed on the surface of merozoites, is one of the well characterized antigens of *P. falciparum*. During late schizogony, MSP-1 is proteolytically processed from its ~190 kDa precursor into four major cleavage products: p83, p30, p38, and p42 [18] designated as MSP-1_83_, MSP-1_30_, MSP-1_38 _and MSP-1_42 _respectively. During erythrocyte invasion, the MSP-1_42 _fragment is further cleaved into MSP-1_33 _and MSP-1_19 _which is essential for invasion (Figure 1A) [19]. The proteolytically processed MSP-1 appears to exist in association with the processed products of MSP-6 and MSP-7 (Figure 1B) [20-22]. Major biochemical and immunological parameters of this multipartite have been described recently [23].

Humoral immune responses to MSP-1 protein subunits, especially, MSP-1_42 _and MSP-1_19 _fragments, are known to be protective against *P. falciparum *infection and clinical malaria [24-33]. Antibodies specific for these antigens have been shown to inhibit both erythrocyte invasion and parasite growth in vitro [26,27]. In some studies, antibody responses to MSP-1_19 _were correlated with clinical immunity to *P. falciparum *[29,30,34] and with reduced parasitaemia and fever [31]. In addition, presence of several T-cell epitopes within the MSP-1_42 _fragment were identified [35] and these epitopes may provide T- helper function needed for the production of anti-MSP-1 antibodies.

Studies of the *msp-1 *gene sequence obtained from different *P. falciparum *isolates demonstrate significant antigenic diversity comprising highly conserved, dimorphic and variable regions. There are two major allelic forms of MSP-1 belonging to either the K1, (as in the FCB-1 strain, here referred to as F allelic form) or the MAD20 (as in the 3D7 strain, here referred to as D allelic form) allelic families [36,37]. Therefore, one would postulate that naturally exposed individuals would mount immune responses to different fragments and allelic forms of MSP-1. However, only a few field studies have investigated humoral responses to both of these allelic forms of the four major subunits of MSP-1 and their associated proteins, MSP-6_36 _and MSP-7_22_[25,38-40] in humans naturally exposed to malaria. A complete characterization of humoral immune responses to this complex protein is therefore required to determine their role in protective immunity and pathogenesis.

The potential adverse effect of malarial antibody responses, including antibodies to the C-terminal region of MSP-1, in the manifestation of CM have been implicated in some studies [41-45]. For example, higher levels of IgM and IgG antibodies to glycosylphosphatidylinositols (GPI) were associated with CM and death in young children in a study conducted in Mali [45]. On the contrary, in another study, reduced anti-GPI antibodies were found in CM patients compared to mild malaria patients [41]. In a Ghanaian study, IgG2 and IgG4 antibody responses to a recombinant *P. falciparum *RIFIN antigen, RIF-29, were exclusively shown to be associated with CM, suggesting that these antibodies might be involved in CM pathogenesis [44].

In the current study, the antibody response to the two major allelic forms (F and D) of MSP-1 antigens (MSP-1_83_, MSP-1_30_, MSP-1_38 _and MSP-1_42_) and their associated proteins, MSP-6_36 _and MSP-7_22_, was systematically characterized in an Indian cohort and the responses compared between two different group of malaria patients and healthy controls.

## Materials and methods

### Study area

The blood samples for this study came from a subset of 121 subjects enrolled in a cohort study designed to assess the severe outcomes associated with CM in the Jabalpur Province of Madhya Pradesh state in India. The study sites included Nethaji Subash Chandra Bose (NSCB) Hospital (a referral hospital for the region) in Jabalpur and Civic Hospital (primary hospital) in Maihar, Satna District. This study was approved by the ethical committees of Morehouse School of Medicine, National Institute of Malaria Research in India, Centers for Disease Control and Prevention (CDC) and the National Institute of Health (NIH) in the USA. Jabalpur province is a malaria endemic region with seasonal transmission of malaria accounting for about 23% of all the malaria cases in Madhya Pradesh state [46]. Previous community based studies conducted in this area revealed that malaria affects all age groups with the highest prevalence occurring in children between 8–14 years of age [47].

### Study details

Three categories of subjects, namely cerebral malaria (CM) patients, mild malaria (MM) patients and healthy control (HC) subjects were enrolled in the study after the informed consent was obtained. Patients from all ages and both sexes who met the study criteria were enrolled. The presence of *P. falciparum *parasitaemia was determined by microscopic examination of thin and thick smears. Clinical histories and information were collected for each patient from physician's records and study questionnaires. The following criteria to enroll the subjects into the different clinical groups were used:

#### Cerebral malaria (CM)

CM was defined as unrousable coma (a non-purposeful response or no response to a painful stimulus) with microscopically diagnosed *P. falciparum *and have no other clinically evident cause of impaired consciousness such as hypoglycemia, meningitis, and encephalitis following WHO criteria [48]. Patients with mixed infection of malaria parasites were excluded.

#### Mild malaria (MM)

Patients who had fever with *P. falciparum *parasitaemia of < 25,000 parasites/μl of blood with no evidence of impaired consciousness or seizures at the time of enrollment with no other past history of mental illness, meningitis or accidental head injury were included.

#### Healthy control (HC)

HC were recruited from relatives of the patients or persons residing in the same study area who did not have malaria infection (as determined by microscopy) or any other febrile illness at the time of enrollment.

### Blood collection

Venous blood samples were collected from children (5 ml) and adults (10 ml) into Becton-Dickinson cell preparation tubes (catalogue #362753, BD Pharmingen, Franklin Lakes, NJ, USA). The blood was centrifuged for 20 minutes at 1500 relative centrifugal force (rcf) to separate plasma. Plasma samples were aliquoted and frozen immediately in liquid nitrogen or at 80°C until use.

#### Plasmodium falciparum antigens

Relatively pure recombinant antigens of the major processing products of MSP-1 [MSP-1_83_, MSP-1_30_, MSP-1_38 _and MSP-1_42 _belonging to both the K1 allelic form (as in the FCB-1 strain, here referred to as F), and the MAD20 allelic form (as in the 3D7 strain, here referred to as D)] together with MSP-1_19 _(3D7), MSP-6_36 _(3D7) and MSP-7_22 _(3D7) were used. These antigens were prepared as described previously [23,49,50] and the purity of the products that were used in this study are shown in Figure 1C.

### Enzyme-linked immunosorbent assay (ELISA)

Microtiter Immulon-2 plates were coated overnight with the MSP complex antigens (500 ng/ml) in phosphate buffered solution at 4°C. Total IgG antibodies were measured using a HRP-conjugated mouse anti-human IgG antibody at 1:8000 dilution (Southern Biotech, Birmingham, AL). IgG subclasses were measured using mouse anti-human IgG subclass antibodies IgG1 (HP6069) at 1:2000, IgG2 (HP6002) at 1:6000, IgG3 (HP6047) at 1:50,000 and IgG4 (HP6023) at 1:20,000 dilution followed by goat anti-mouse IgG-HRP at 1:2000 dilution (Southern Biotech, Birmingham, AL). Samples were tested at a 1:400 dilution and 1:100 dilution for total IgG and subclasses respectively. Ten pooled plasma samples from malaria naïve donors from USA were selected and used as negative controls in each experiment. In addition, each ELISA plate contained a duplicate of blank wells with no plasma sample and the mean background OD value obtained from these wells was subtracted from all other experimental wells. Test samples were considered positive when the OD values were higher than the mean OD plus two standard deviations of pooled negative control sera. The percentage prevalence was calculated as: (total number of positive sera/total number of sera tested) × 100.

### Statistical analysis

ELISA absorbance values (measured as optical density, OD) were compared and analysed by a non-parametric test (Kruskal-Wallis test). Differences in the percentage prevalence of the antibodies were calculated using a multiple comparison analysis controlling for age and parasitaemia. Antibody levels for the different subgroups of MSP-1 antigens (D and F) were assessed for correlation by Spearman's rank correlation. All statistical tests were performed using STATA™ 8.2 (College Station, TX, USA) and SAS (9.1, NC, USA).

## Results

### Study samples

The characteristics of subjects who participated in this study are summarized in Table 1. Among the 121 samples tested for total IgG, 16% were from patients in the HC category, 38% from MM and 46% from CM. Of the 120 samples tested for the subclasses, 15% were from HC, 41% from MM and 44% from CM (Table 1).

**Table 1:** Characteristics of the study participants

	**HC**	**MM**	**CM**^a^
Total No. of samples for IgG	19	46	56
Total No. of samples for subclasses	18	49	53
No. of males used for total IgG	5	20	18
No. of males used for subclasses	4	28	36
No. of females used for total IgG	14	26	38
No. of females used for subclasses	14	21	17
Median age for samples used for total IgG (yr)	30.0	18.5	22.0
Median age for samples used for subclasses (Yr)	27.0	19.0	22.0
Median parasitaemia (parasite/μl) of samples used for total IgG	N/A	1,507	960
Median parasitaemia (parasite/μl) of samples used for subclasses	N/A	1,413	987

The demographic and parasitological characteristics of the study participants are shown. A total of 121 samples were used for the total IgG assay and 120 for the IgG subclasses assay. HC = healthy controls, MM = mild malaria and CM = cerebral malaria. Seven patients in the CM group died of CM. Both age and parasitaemia data are represented as median. ^a ^CM patients include seven patients who died of CM

### Prevalence and levels of total IgG antibodies to various antigens of MSP-1 complex

**Figure 2:**
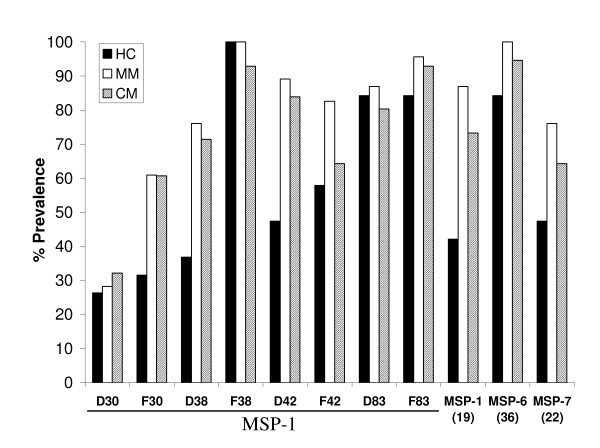
**Prevalence of anti-MSP total IgG for the different subunits**. The total IgG antibody response to the various MSP-1 subunits, MSP-6_36 _and MSP-7_22 _was measured by ELISA. The percentage prevalence was calculated as follows; (total number of positive sera/total number of sera tested) × 100. The prevalence of total IgG antibodies was statistically different between HC and MM for the indicated antigens (* = P < 0.05).

As shown in Figure 2, total IgG antibody prevalence to the different antigens varied greatly. Overall, the highest prevalence of IgG response was seen in the MM group followed by the CM group and the HC group, the latter showing the lowest prevalence with a few exceptions. However, these differences were significantly different between HC and MM groups only for MSP-1_d38 _(P < 0.006), MSP-1_d42 _(P < 0.003) and MSP-1_19 _(P < 0.01) antigens. MSP-1_d30 _and MSP-1_f30 _showed the lowest responses while the MSP-1_f38_, MSP-1_d83_, MSP-1_f83 _and MSP-6_36 _antigens showed the highest responses. The F allelic forms of MSP-1_30 _and MSP-1_38 _demonstrated higher responses compared to the D allelic forms.

**Figure 3:**
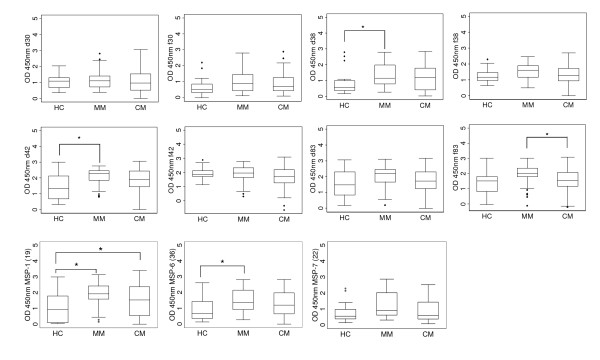
**Levels of total IgG antibody responses to MSP-1 subunits, MSP-6_36 _and MSP-7_22 _antigens**. Total IgG antibody responses were measured. Box plots depict median values with 25th- and 75th-percentile values represented by the bottom and top edges of boxes. Small diamonds indicate values that fall outside of the error bars. Only a few antigens demonstrated statistically significant differences (* = p < 0.05) when the antibody levels were compared among the different disease categories. The different subunits and allelic forms of MSP-1 are represented as d30, f30, d38, f38, d42, f42, d83 and f83.

The mean antibody levels reported in Figure 3 illustrate important differences in the IgG antibody levels between the three groups. Significantly higher levels of antibody responses in the MM group compared to the HC group were observed for MSP-1_d38_, MSP-1_d42_, MSP-1_19 _and MSP-6_36 _antigens (P < 0.05). The CM and the HC group had similar levels of antibodies except in the case of MSP-1_19_, which showed higher levels of antibody responses in the CM group compared to HC group (P < 0.05). Although there was a trend towards lower levels of antibody responses in CM as compared to the MM group for several antigens, the difference was significant only for MSP-1_f83 _(P < 0.05).

### IgG subclass responses to MSP complex antigens

**Figure 4:**
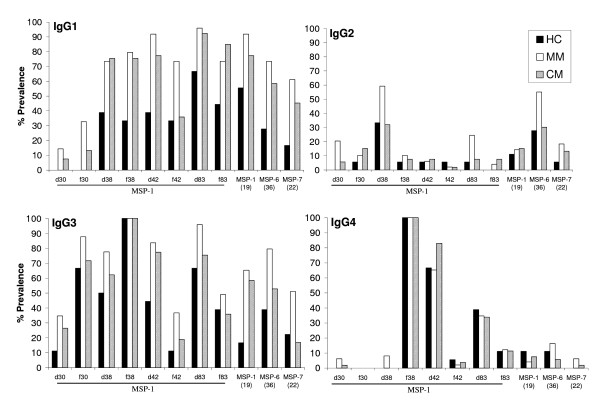
**Prevalence of anti-MSP IgG subclasses for the different subunits**. The prevalences of the four IgG subclasses are shown. Test samples were considered positive if their OD values were higher than the mean plus two standard deviations of 10 pooled plasmas from malaria naïve donors from North America. The percentage prevalence was then calculated as described in figure 2.

As previously reported [51], a skewing towards IgG1 and IgG3 subclasses in the prevalence and levels of antibody responses to most of the antigens was observed. IgG2 and IgG4 responses were generally very low with a few exceptions (Figures 4). Interestingly, all the plasmas were positive for anti-MSP-1_f38 _IgG3 and IgG4 (100% prevalence) regardless of disease category (Figure 4). As observed with total IgG responses, the MM group showed higher prevalence of IgG1 responses to eight antigens and IgG3 responses to for five antigens compared to HC. The CM group showed higher prevalence of IgG1 responses to three antigens and higher IgG3 responses to two antigens as compared to the HC group. Significantly lower prevalence of anti- MSP-1_f30 _and MSP-1_f42 _IgG1 (p < 0.05), anti- MSP-1_d83_, MSP-6_36 _and MSP-7_22 _IgG3 (p < 0.05) and anti- MSP-1_d38 _and MSP-6_36 _IgG2 (p < 0.05) were observed in CM patients compared to MM patients. The prevalence of IgG2 antibody responses was below 20% for all antigens except for MSP-1_d38_, MSP-1_d83 _(in MM cases) and MSP-6_36_. Only MSP-1_f38_, MSP-1_d42 _and MSP-1_d83 _antigens showed IgG4 antibody in more than 20% of the patients and there were little differences in the prevalence of IgG4 responses among the three patient categories.

**Figure 5:**
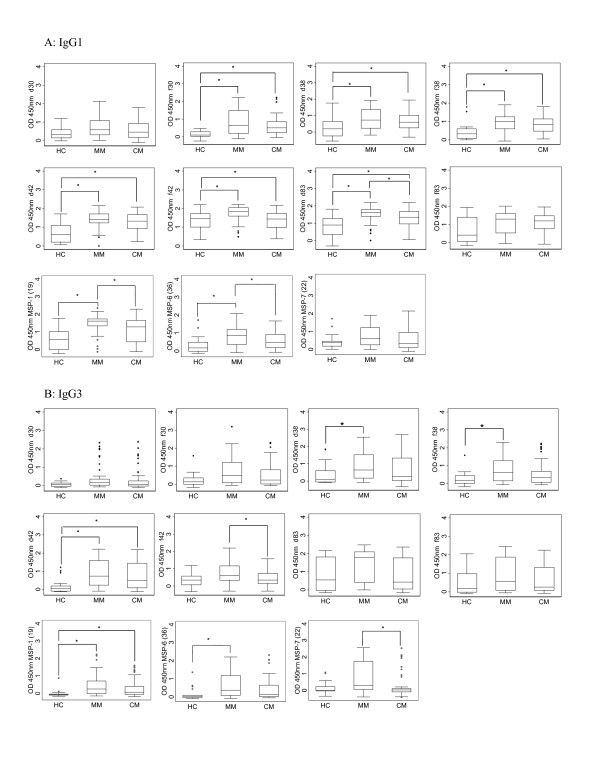
**Levels of IgG subclasses antibody responses to MSP-1 subunits, MSP-6_36 _and MSP-7_22 _antigens**. The IgG1 (A) and IgG3 (B) antibody responses to the two allelic forms of each of the MSP-1 subunits, MSP-6_36 _and MSP-7_22 _were measured. The box plots depict median values with 25th- and 75th-percentile values represented by the bottom and top edges of boxes. Small diamonds indicate values that fall outside of the error bars. Statistically significant differences are depicted with the * (p < 0.05).

The differences in the median of antibody levels (IgG1 and IgG3) are reported in Figure 5. IgG2 and IgG4 responses were not included as there were only negligible amounts of antibodies present. Some antigens, such as MSP-1_f42_, MSP-1_f83_, MSP-1_19 _and MSP-7_22_, evoked more IgG1 antibodies than IgG3 while MSP-1_d30_, MSP-1_f30 _and MSP-1_f38 _elicited more IgG3 antibodies than other subclasses. Significantly higher levels of IgG1 for 8 antigens and IgG3 for five antigens antibody levels were found in MM patients compared to HC. The CM group showed higher levels of IgG1 for six antigens (Figure 5A) and IgG3 for two antigens as compared to HC (Figure 5B). Significantly lower antibody levels in CM patients compared to MM patients for anti- MSP-1_d83_, MSP-1_19 _and MSP-6_36 _IgG1 antibodies (P < 0.05) (Figure 5A) and anti- MSP-1_f42 _and MSP-7_22 _IgG3 antibodies (P < 0.05) (Figure 5B) were observed.

### Correlation of the two dimorphic alleles of MSP-1 antigens for total IgG

**Figure 6:**
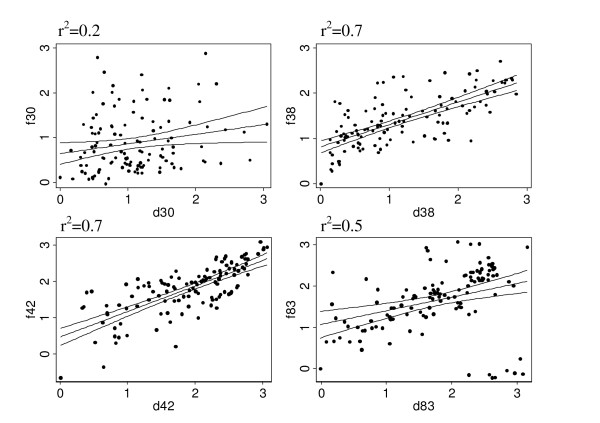
**Correlation of antibody responses between the two dimorphic alleles, MSP-1D and MSP-1F**. To determine if there was a correlation in antibody responses between the two allelic forms of MSP-1, a scatter plot of anti-MSP-1D versus MSP-1F total IgG antibody responses was generated. The lines represent the fitted values and the 95% confident interval lines. The Spearman correlation coefficients are shown in the upper left corner.

In order to examine to what extent the two alleles are related to each other in terms of eliciting antibody responses, correlations in antibody responses between the two allelic forms were determined. In general, there was a strong correlation in the IgG antibody responses between MSP-1_38_,, MSP-1_42 _and MSP-1_83 _allelic forms. The correlation was lowest for the MSP-1_30 _antigen (Figure 6). A similar result was observed for IgG1 (p30; r^2 ^= 0.4, p38; r^2 ^= 0.7, p42; r^2 ^= 0.6, p83; r^2^= 0.4) and IgG3 (p30; r^2 ^= 0.5, p38; r^2 ^= 0.7, p42; r^2 ^= 0.6, p83; r^2 ^= 0.7) antibody responses.

## Discussion

In this study the differences in antibody responses to MSP complex proteins among CM patients, MM patients and HC subjects in a malaria-endemic part of India was investigated. This is one of the first studies to address systematically the antibody responses to the two major allelic forms of all the four major subunits of MSP-1 antigens (MSP-1_30 _MSP-1_38 _MSP-1_83_, MSP-1_42_) together with their associated proteins, MSP-6_36 _and MSP-7_22 _in malaria patients. The HC group tended to have lower antibody levels than MM and CM patients consistent with previous studies, which demonstrated that antibodies to merozoite antigens were higher in parasitaemic compared to aparasitaemic subjects [38,52-54]. An important finding is the observation that CM patients showed significantly lower antibody responses to some of the MSP family of antigens as compared to MM patients. These differences included both low prevalence and low mean IgG levels and IgG subclasses in the CM group. On the contrary, MM patients showed significantly elevated IgG responses to many of the antigens compared to the HC group.

The association of lower antibody titers to certain *P. falciparum *antigens with malaria severity (such as CM) has been demonstrated in previous studies. For example, significantly lower levels of *P. falciparum *anti-GPI IgGs were observed in CM patients as compared to MM patients in a study in Senegal [41]. In the Senegal study, the differences in the responses to MSP-1_19 _antigen were slightly lower in the CM patients compared to the MM patients, although significantly lower levels were only observed in the CM non-survivors sub-group. In the current study, antibody responses to the MSP-1_19 _antigen and some other MSP complex antigens were lower in CM patients as compared to MM patients. On the contrary, other studies have reported higher levels of anti- GPI antibodies in CM patients compared to non-severe malaria patients or HC subjects [45]. In another study, higher levels of IgG2 and IgG4 antibody responses to the variant surface glycoprotein RIF-29 were found exclusively in CM children but not in the non-cerebral malaria controls [44]. Overall, findings from the current study are consistent with the hypothesis that CM patients may have some deficiency in mounting optimal antibody responses to some antigens essential for clinical protection. However, additional evidence to confirm this hypothesis is required and validation will depend on further studies.

The seropositivity of antibodies varied considerably depending on the MSP antigen as previously demonstrated [39]. Antibody responses were relatively high for most of the antigens, which was not surprising given that this study was conducted in a malaria endemic region where the majority of people are exposed to malaria. Both MSP-1_42 _and MSP-1_83 _demonstrated high antibody prevalence compared to the other fragments, consistent with a previous study [39]. Anti-MSP-6_36 _antibodies were shown to be generated in individuals naturally infected with *P. falciparum *[55]. Additionally, these antibodies were thought to play a role in the inhibition of erythrocyte invasion [55,56] and parasite multiplication [23,57]. This study demonstrates that naturally exposed residents in central India also generate both anti-MSP-6_36 _and anti-MSP-7_22 _antibodies. In contrast, the antibody prevalence to the MSP-1_d30 _antigen was low as had been observed in previous studies [39], suggesting that this may be a poorly immunogenic antigen.

Results from this study confirm that the entire MSP-1/MSP-6/MSP-7 complex contains B-cell epitopes capable of generating specific antibodies in naturally exposed individuals.

The F allelic form of MSP-1_30 _and MSP-1_38 _antigens showed higher seroprevalence than the corresponding D allelic forms, suggesting that individuals generate specific antibodies to the different allelic forms of these antigens. The higher prevalence of antibodies to the F allelic forms of MSP-1_30 _and MSP-1_38 _antigens may be due to a predominant presence of *P. falciparum *parasites with this allele in this population in India. However, there is no published information to verify the relative proportions of these alleles within this population. A few studies that have investigated the prevalence of the different MSP-1 alleles in India have either demonstrated the predominance of the MAD20 allele (D), as defined by sequence analysis of the 16^th ^and 17^th ^block [58], or no bias to any allele [59,60].

However, the MSP-1_42 _and MSP-1_83 _antigens showed similar antibody prevalence to the two allelic forms of the antigens, suggesting the development of antibodies cross reactive to both allelic forms. Significant correlations in the antibody levels elicited by the two allelic forms of MSP-1 were observed except for the p30 fragment. The MSP-1_30 _is located within the dimorphic region of the MSP-1 protein [36]. Therefore, different epitopes in these dimorphic regions may be presented to B-cells generating heterogeneity in antibody responses between the two alleles. These results demonstrate that, at least for p38, p42 and p83, responses to one allelic form predict positive responses to the other allelic form. It is possible that conserved epitopes between the two allelic forms are presented to the immune system leading to the generation of cross-reactive antibodies. In fact, a previous study demonstrated a high degree of cross-inhibition between antibodies generated against the D and the F allelic forms of MSP-1 [39].

IgG subclass analysis showed that IgG1 and IgG3 were the predominant subclasses for most of the antigens studied, consistent with previous studies [34,51,61]. While mixed IgG1/IgG3 responses to all MSP antigens used was detected, the relative proportions of these two subclasses were different for the different MSP antigens, implying that IgG class switching may be greatly influenced by the characteristics of the antigen as previously suggested [40,51,62]. It has been suggested that conserved antigens, such as MSP-1_19_, typically induce IgG1, while highly polymorphic antigens induce IgG3 [63]. Interestingly in this study, such a correlation was not observed except for the MSP-1_19 _antigen which elicited more IgG1 than IgG3 as previously observed [40,51,61,64]. In contrast, a mixed response to the conserved MSP-6_36 _was observed while a previous study in southern-central Vietnam demonstrated a skewing towards IgG1 [63]. Similarly, predominant IgG3 responses were observed for the highly polymorphic block 2 region (found within the p83 subunit) of MSP-1 in an African population [40] and for polymorphic MSP-7_22 _in Vietnam [63]. However, in the current study, MSP-7_22 _induced more IgG1 antibodies than IgG3 and MSP-1_d83 _showed a mixed response, while MSP-1_f83 _elicited more IgG1 antibodies than IgG3. These differences may be due to differences in innate characteristics of the host population or epidemiologic differences.

## Conclusion

Observations from the current study provide insight into the intricate pattern of acquired humoral responses to the MSP-1/MSP-6/MSP-7 complex. The results demonstrate that MSP-1/MSP-6/MSP-7 complex antigens are immunogenic in humans naturally exposed to *P. falciparum *infection in India, supporting the view that full size MSP-1 is a particularly interesting vaccine candidate. In addition, this study suggests that there may be a dysregulation in the generation of optimal antibody responses to some of the MSP-1 protein fragments in CM patients and it remains to be determined if such differences contribute to susceptibility of individuals to CM.

## Competing interests

The authors declare that they have no competing interests.

## Authors' contributions

NWL: Participated in the study design, performed the experiments and data analysis and wrote the manuscript. JET: Participated in the study design, data analysis and editing of the manuscript. VJ: Participated in the cohort study design, enrolled patients, collected clinical and epidemiologic data and biological samples and helped in the editing of the manuscript. ACN: Participated in designing the cohort, enrollment of subjects, clinical evaluation and editing of the manuscript. CWK: Participated in the expression and purification of the recombinant proteins, study design and assisted in editing the manuscript. UW: Participated in the expression and purification of the recombinant proteins, study design and in the editing of the manuscript. HB: Facilitated the expression and purification of the recombinant proteins, planning of the study and contributed to the editing of the manuscript. APD: Participated in planning of the study, supervision of the study in India and editing of the manuscript. NS: Participated in the cohort study design, enrollment of study subjects, planning of the experiments and editing of the manuscript. JKS: Participated in the cohort study design, planning of experiments and assisted in the editing of the manuscript. VU: Participated in the study design, planning of the experiments, data analysis, and contributed to manuscript writing and editing.
